# Quantifying the effects of climate and anthropogenic change on regional species loss in China

**DOI:** 10.1371/journal.pone.0199735

**Published:** 2018-07-25

**Authors:** Jinxing He, Chuan Yan, Marcel Holyoak, Xinru Wan, Guoyu Ren, Yangfang Hou, Yan Xie, Zhibin Zhang

**Affiliations:** 1 Institute of Zoology, Chinese Academy of Sciences, Chaoyang District, Beijing, P. R. China; 2 University of Chinese Academy of Sciences, Shijingshan District, Beijing, P. R. China; 3 Department of Environmental Science and Policy, University of California Davis, Davis, CA, United States of America; 4 Department of Atmospheric Science, School of Environmental Studies, China University of Geosciences, Hongshan District, Wuhan, P. R. China; 5 National Climate Center, China Meteorological Administration, Haidian District, Beijing, P. R. China; 6 Center for Historical Geographical Studies of Fudan University, Yangpu District, Shanghai, P. R. China; Sichuan University, CHINA

## Abstract

Human-induced environmental and climate change are widely blamed for causing rapid global biodiversity loss, but direct estimation of the proportion of biodiversity lost at local or regional scales are still infrequent. This prevents us from quantifying the main and interactive effects of anthropogenic environmental and climate change on species loss. Here, we demonstrate that the estimated proportion of species loss of 252 key protected vertebrate species at a county level of China during the past half century was 27.2% for all taxa, 47.7% for mammals, 28.8% for amphibians and reptiles and 19.8% for birds. Both human population increase and species richness showed significant positive correlations with species loss of all taxa combined, mammals, birds, and amphibians and reptiles. Temperature increase was positively correlated with all-taxa and bird species loss. Precipitation increase was negatively correlated with species loss of birds. Human population change and species richness showed more significant interactions with the other correlates of species loss. High species richness regions had higher species loss under the drivers of human environmental and climate change than low-richness regions. Consequently, ongoing human environmental and climate changes are expected to perpetuate more negative effects on the survival of key vertebrate species, particularly in high-biodiversity regions.

## Introduction

Our planet is facing an accelerated decline in global biodiversity, which is threatening ecosystem functioning, ecosystem services, and human wellbeing [1]. Global species extinction rates have rapidly increased during the past 200 years, to much higher levels than during the pre-human period [2–6]. Of all taxa, terrestrial vertebrates are known to have suffered the largest species loss or threat of extinction from human environmental change[4, 5] and climate change[5–8], and continue to experience rapid decline in both population abundance[4] and size of their geographic range[5, 6].

The current mass extinction crisis, also named as the “sixth extinction wave,” was clearly triggered by humans[9]. Human population growth and economic development significantly increased the human effects on wildlife through environmental change and disturbances such as habitat destruction, pollution, impacts from invasive species, and primarily human-introduced pathogens; all these factors have been implicated in biodiversity loss [4, 10–12].

Climate change is also expected to impact terrestrial biodiversity, including species-level reductions in range size and abundance [8]. Past climate conditions have played a role in shaping the present distribution of species, often through species-specific physiological thresholds of temperature and precipitation tolerance[13]. Geographical variation in climate is strongly correlated with biodiversity and species richness patterns [14] of terrestrial vertebrates. Climate change has been implicated in poleward and upward range shifts of many taxa during the twentieth century[15], which may result in local or regional extinction. According to a meta-analysis of 131 studies, the estimated percent of species predicted to go extinct from climate change varied from 0 to 54%, with an average of 7.9% [16]. Hence, on average, extinction risks are expected to increase with future climate change.

Many studies indicate high biodiversity systems or regions are more resistant to external disturbances [17, 18]. Positive interaction among species via facilitation is often more common in communities with higher species richness[19]. However anthropogenic factors may increase species loss through altering species interactions, such as plant-pollinator interactions[20], plant-frugivore interactions[21], host-parasitoid interactions[22] and food web interactions [23, 24]. Ultimately, if anthropogenic change eliminates the organisms that are essential to a species then that species may be driven extinct [20]. Therefore, species loss driven by human disturbance, environmental change, or climate change could cause a cascade of species extinctions in high diversity regions [22–24].

Estimation of the proportion of species lost is essential for quantifying the effects of anthropogenic change and disturbance and taking action to improve management and conservation to protect biodiversity. Currently, there is still high uncertainty in estimating species loss or extinction rates due to lacking of reliable direct methods or data[25]. Species loss or extinction rates are mostly estimated indirectly based on species-area relationships, Red Data lists, or climate change models, but debate on these estimates is ongoing because of the wide variation in observed extinct rates [25–27]. Currently, there are few direct estimates of rates of species loss at small spatial scales despite increasing trend of anthropogenic change and disturbance[4, 28]. During past half century, we faced increasing trend of both human disturbance and climate warming, thus it is necessary to differentiate between human disturbance and climate change as the causes of diversity loss.

China is one of the mega-diversity countries of the world and home to many globally valued species that are conservation priorities[29]. China is also the most populous country and has a very large diversity of environments. China's biodiversity is under severe threat due to accelerated human activities, rapid economic growth and clear climate change during the last half century [30]. In the 20^th^ century, seven Chinese vertebrate species became extinct or extinct in the wild. Of 4357 vertebrate species distributed in China, 932 species were listed as highly threatened by IUCN [31]. Of the 174 endangered species, 118 of them showed population declines between 2000 and 2013[32].

In this study we estimated the proportion of species lost across 2,365 counties in China between 1950 and 2000. We used county-level species occurrence data for 252 protected vertebrate species. To understand the effects that human disturbances and climate change on these changes, we quantified the associations of species loss rates with human population density, climatic variables and species richness at the county level. Human population density is a proxy for environmental change and direct human disturbance and is known to be correlated with other global change variables such as rates of habitat loss and overexploitation.[33, 34]

## Methods

### Species occurrence data

We used two available data sources of the key protected species of terrestrial vertebrates in China. The first dataset of the number of species in each county-level region during 1997–2000 was extracted from a report from a national survey by the State Forestry Administration [35]. Occurrence for the 252 key protected species in 2356 counties of mainland China was compiled from this book. We assumed that the species was lost in the county if it was not reported in this book. The second dataset described the number of species in each county-level region was compiled using various published sources since 1950[36]. This database has the known maximum species occurrence records of each county-level region since 1950. Species with unclear county-level resolution of distribution data were excluded from analyses. The county-level regions were defined based on the administrative division of mainland China in 1999[37]. There are 2,365 county-level regions (407±1026 km^2^) from all 31 provinces, municipal cities and autonomous regions (no data for regions of Hong Kong, Macao autonomous regions and Taiwan province) in mainland of China. The estimated percent species lost (PSL) in each county until 2000 comparing with the maximum record of its past 50 years was calculated as follows:

$$PSL=(N_{0}-N_{1})/N_{0}\times 100\%$$

Where, for each county, N_1_ is the number of known surviving species during 1997 to 2000, and N_0_ is the maximum known number of species from 1950 to 2000.

### Testing the effects of human disturbance, climate change and species richness

**Fig 1:**
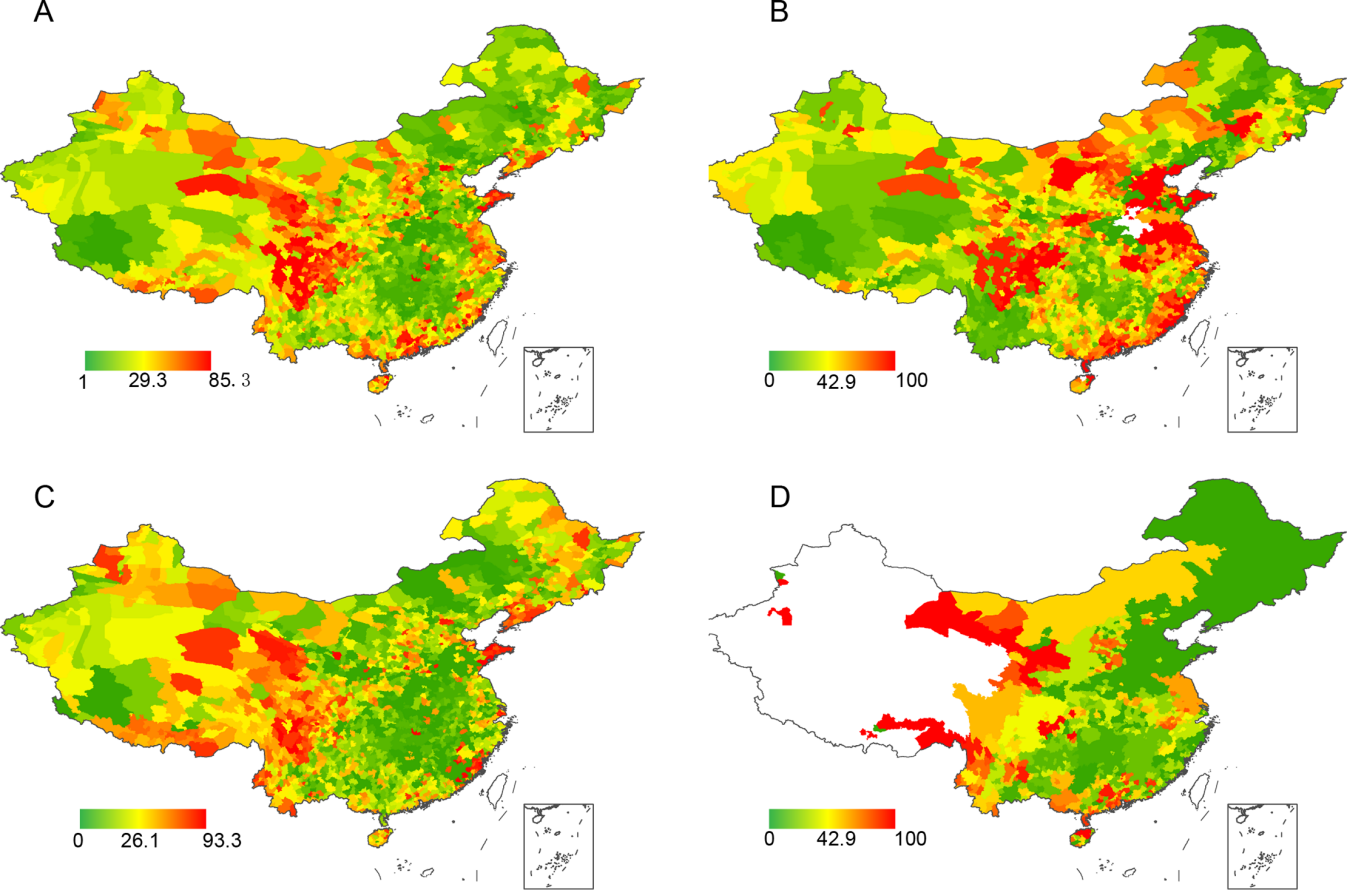
**Percent of species lost (PSL) in 2,365 counties in China for all species (A), mammals (B), birds (C), and amphibians & reptiles (D).** The color range indicates PSL; empty area indicates missing values.

We used the difference between the mean annual temperatures from 1951 to 1955 and from 1996 to 2000 in each county to represent change of temperature (T) during the last half century. We used the difference between the mean annual precipitation from 1961 to 1965 and from 1996 to 2000 to represent change of precipitation (P). We used the difference between the human population density in 1953 and 2000 to represent the change of population density (H), a correlate of environmental change and disturbances. We used latitude and longitude of the centroid of each county to account for spatial autocorrelation in statistical models [38]. The mean annual temperature (in 0.1°C) during 1950–2000 and mean annual precipitation (in millimeter) during 1960–2000 were extracted from the National Meteorological Information Center (NMIC) of China[39]. The human population density in each county in 1953 and 1999 was obtained from the Chinese Population Geographic Information System[40]. We assigned the data into grid cells with a size of 0.001×0.001° latitude-longitude and calculated the mean values of these cells for each county based on the Administrative division of mainland China in 1999 in ArcGIS (version 10.3). We used the number of species for different classes of all terrestrial vertebrates in each county as the species richness (B) using our second species dataset described above [36]. The percent of species lost and species richness for all species (PSL and B), mammals (PSL_m_ and B_m_), birds (PSL_b_ and B_b_), and amphibians and reptiles (and B_a_), human population density change (H), temperature change (T) and precipitation change (P) were illustrated by using ArcGIS (Fig 1, S1 and S2 Figs, S1 Dataset) (version 10.3). We used Analysis of Variance (ANOVA) to test the differences of percent of species lost of all species, mammals, birds, and amphibians and reptiles.

### Statistical analysis

Firstly, we used Generalized Additive Models (GAMs) to investigate the main effects of human population change, climate change and species richness on the proportion of species lost (PSL/100) for all species, mammals, birds and amphibians-reptiles at the county level in China. The initial model was built as follows:

PSL/100=a×T+b×P+c×H+d×B+s(cent.x,cent.y)

Because the response variable PSL/100 was a proportional variable, it was modeled with a binomial distribution. Constants *a*, *b*, *c* and *d* were regression coefficients of the variables, and *s*(cent.x, cent.y) is the 2d-smoothing function modeling the spatial autocorrelation effects where cent.x and cent.y is the centroid longitude and latitude of a county. The best-fitting models were selected using the lowest value of AICc, in making statistical inference. We checked the spatial autocorrelation of residuals from GAMs to validate the robustness of the model results (S3 Fig).

Secondly, we tested the linear interactive effects between variables. The model structure and selection were similar to the above model except that pairwise interactive terms of variables that showed significant main effects in previous analyses were included in each model. All the analyses were performed by *mgcv* and *MuMIn* package in R software.

## Results

Our data indicate that, during the past half century, middle-east and south-east China experienced a large increase of human population density (S1A Fig), northwest and northeast China experienced a large temperature increase (S1B Fig), and south and west China showed a large increase of precipitation (S1C Fig). Several regions showed a comparatively large species loss, particularly in west China and coastline provinces which experienced large population or temperature increase (Fig 1).

The percent of species lost (PSL) of the 252 key protected vertebrate species at the county level in China was 27.2% within the approximately 50 years period. The average PSL of mammals (47.74%) was greater than that of amphibians and reptiles (28.8%), and in turn greater than that of birds (19.75%; Table 1).

**Table 1:** The average percent of species lost (PSL) ± standard deviation of 252 key protected vertebrates of 2,365 counties in the mainland of China from approximately 1950 to 2000. a, b, and c indicate significantly different taxon groups (*p* < 0.05, ANOVA).

	Mammals	Birds	Amphibians and reptiles	All species
Average PSL ±SD	47.74±33.59^a^	19.75±17.70^b^	28.80±28.86^c^	27.21±16.26^c^

Temperature increase was positively related to species loss of all species and birds but not mammals or amphibians and reptiles (Table 2). Precipitation increase was negatively related to species loss of birds but not the other taxa. Both human population increase and species richness showed significant positive effects on species loss of all species, mammals, birds, and amphibians and reptiles (Table 2).

**Table 2:** Main effects of temperature change index (T), precipitation change index (P), human population density change index (H), species richness (B) on the proportion of species lost of all vertebrates, mammals, birds and amphibians and reptiles (R&A) from the 252 key protected species in mainland of China.

	T	P	H	B	R^2^	AICc	Weight
All	3.29**		5.25***	24.79***	0.434	12097.5	0.649
Mammals			5.56***	5.99***	0.344	8342.5	0.486
Birds	3.24**	-2.75**	4.31***	27.70***	0.451	8847.6	0.881
R&A			1.90*	4.85***	0.496	4693.3	0.337

Values in the tables are Z-values, and

*** P< 0.001

** P<0.01, and

* P<0.05.

As shown in Table 3, there were significant positive interactions between the effects of temperature increase and species richness on proportion of species lost for all species and for birds but not the other taxa. The positive interactive effect between human population increase and species richness on PSL was significant for all taxa, mammals, and amphibian and reptiles, but not birds. Precipitation increase showed a positive interactive effect with species richness, but a negative interactive effect with human population increase on proportion of species lost of birds. There was a positive interactive effect between temperature increase and human population increase for all taxa.

**Table 3:** Linear interactive effects between temperature change (T), precipitation change (P), human population density change (H), species richness (B) on the proportion of species lost of all vertebrates, mammals, birds and amphibian and reptile (R&A).

	T	P	H	B	T×B	P × B	H × B	T × H	P × H	R^2^	AICc	Weight
All	-1.50		-0.88	8.11***	9.23***		2.63**	2.56**		0.445	12004.2	0.785
Mammals			0.41	4.49***			4.37***			0.348	8322.0	1
Birds	2.03*	-2.83***	5.40***	11.53***	2.34*	2.52*			-2.90**	0.454	8833.6	0.128
R&A			-0.45	3.93***			2.17*			0.497	4690.6	0.722

Values in the table are Z-values, and

*** P< 0.001

** P<0.01, and

* P<0.05.

## Discussion

### Species loss

Our estimated percentage of species lost of 27.2% within about 50 years for 252 key terrestrial vertebrate species is comparable to an estimate that terrestrial vertebrate species declined in abundance by an average of 25% within 4 decades[4]. Our estimated percent of species lost of 47.7% for mammals is larger than an estimate that 173 declining mammal species from six continents lost over 50% of their historic range area during a 100 year period[5], suggesting mammals suffered higher extinction risk in China. Biodiversity loss often varies between taxonomic groups[2, 41]. Our study found that the county-level species loss of mammals (47.74%) was significantly higher than that of birds (19.75%), while that of reptiles and amphibians (28.80%) was intermediate (Table 1). This rank is consistent with global estimates of species loss in previous studies[2, 3]. Compared to birds, mammals experience a much stronger loss because of habitat fragmentation, infrastructure and risk of hunting [42, 43], which is likely related to the higher mobility of birds compared to mammals [33]. Additionally, birds have a higher ability to recolonize habitat than mammals, such as colonization of restored habitats in urbanized areas[28, 33]. The species loss of reptiles and amphibians is intermediate between those of birds and mammals in our study. As compared with birds, reptiles and amphibians obviously have a lower mobility and ability to avoid human disturbance or recolonize their habitat after local extinctions. However, previous studies indicate that amphibians may be less affected by habitat fragmentation[44] and overexploitation[34] than other terrestrial vertebrates. Hence amphibians are less affected by the anthropogenic environmental change and disturbance than mammals.

### Effect of human activities

Humans are considered the major cause of loss of both species and populations of wildlife, causing increased species loss in recent decades[4]. Human population increase is often considered as the ultimate cause of extinction of mammals and birds[45]. Previous studies primarily assessed human impacts on modern biodiversity loss or species extinction rates in qualitative or indirect ways[2, 4, 34]. Our analyses using small-scale spatial-temporal data indicated that counties with high human population increase showed significantly greater proportion of species lost of all species, mammals, birds, and amphibians and reptiles (Table 2). Human population increase was likely an indirect factor in causing biodiversity loss via hunting, pollution and habitat destruction and fragmentation, which have been correlated with local extinction[10, 45]. Our result also provides potential explanations and quantitative evidence why human footprint is often negatively correlated with species richness in every biogeographical region including China[46].

Our results indicated that human environmental change and disturbance, as represented by human population change showed a variety of interactive effects with species richness and climatic factors (temperature and precipitation). Regions with less species richness are often highly modified and human-disturbed habitats including farmlands and cities. The species inhabiting these species-poor habitats are either more resistant to human disturbance or less neophobic than species living in less anthropogenically modified and disturbed habitats with high species richness (e.g. forests or grasslands). This difference may explain the interactive effect of human population change and species richness on species loss. Human activity during the past half of a century has significantly fragmented the habitats of wildlife in China[47]. Animals were likely not able to move freely among habitat fragments under the influence of climate warming, or if they were prone to drought stress (or dehydration). Such affects may explain the significant interaction between human population change and temperature (on all vertebrate species) or precipitation (on birds) in causing local extinction or population decline of species[48].

### Effect of climate change

Many studies have demonstrated range shifts of terrestrial vertebrates in response to changing climate[49]. However, we could find no published quantitative analyses on the effects of temperature or precipitation change on modern biodiversity loss or extinction rates of terrestrial vertebrates. Our study found that birds were the only taxon in which climate change increased proportion of species lost; temperature increase or precipitation decrease showed negative effects on birds’ survival (Table 2). Our results are consistent with previous observations of 7441 terrestrial vertebrate species based on IUCN data, showing that climate change increased the level of imperilment (as indicated by IUCN Red List Criteria) of birds, while mammals, amphibians and reptiles were less affected[34]. Birds can easily shift their ranges northwards or to higher elevations across fragmented habitats driven by current rapid climate warming, which could cause species extinction of populations in their unfavorable habitats. Thus, climate change is expected to be more likely to outpace the response capacity of mammals than birds based on mobility of different taxa [50].

We found climate change also had interactive effects on species loss with species richness (Table 3). The reasons for the interaction are likely similar to those between human population change and species richness described above. Species living in species-poor habitats could well be generalists who are tolerant of human habitat change, potentially making them more resistant to anthropogenic change and disturbances.

### Effect of species richness

A large body of recent and classical ecological research has focused on how biodiversity influences the stability of communities[51]. Species richness is thought to influence community persistence via several mechanisms[52]. A prevalent view is that species-rich communities are more resistant to climate and anthropogenic environmental disturbances than species-poor communities[53]. Our results indicate that biodiversity-high counties or regions showed higher species loss for all taxa (Table 2, Fig 1), not supporting the popular views that more complex ecosystems are more resistant to external disturbances. We believe that this is because of the aforementioned interactive effects between human impacts and climate change.

At present, little is known about how different stressors interact with each other in causing biodiversity loss[11]. Interactions among these factors may amplify the effects on defaunation[11]. Fragmentation would increase accessibility to humans, and then pronounced threats of reduced habitat and exploitation[4, 12]. Extensive land-use change by humans would prevent movement of animals to expand or shift their distributions so as to adapt to habitat changes driven by climate change[4]. In our study, high species richness showed positive interactive effects with human population increase on species loss of all taxa except for birds, with temperature increase and precipitation decrease on birds (Table 3). This suggests that in high-biodiversity regions, China suffered more species losses due to increased human disturbance and climate warming or drought during the past half century. In more complex systems, ecosystems are robust to small external disturbances due to the positive effects of facilitation and complementarity among species[11, 53, 54]. However, if external disturbances are large (like under strong human disturbance, or environmental or climate change), extinction of one species will lead to the extinction dependent interacting species [54]. Many key functional aspects of ecosystems closely depend on biotic interactions, their losses may have pervasive effects in accelerating species local extinction and decay of ecosystem functioning [55]. Positive species interactions are particularly sensitive to different anthropogenic drivers, often shifting their frequency or becoming disrupted due to large environmental changes[56]. Therefore, contrary to the literature cited above complex ecosystems might be more largely altered under strong human disturbances or climate change. In our study, simple ecosystems with less species often represented the highly human-disturbed systems (farmlands, cities etc.). The surviving species in these simple systems might be more resistant to further external disturbances, thus, this may be an alternative explanation on the observed interaction between species richness and human population or climate changes.

### Limitations of this study

Historical records of species occurrences are essential in understanding spatial-temporal patterns of species extinction under the drivers of global change. However, such kinds of data are often inadequate, and would suffer defects of absent or incorrect records. In this study, we calculated the proportion of species lost of 252 terrestrial vertebrates by comparing data between two time periods. It should be pointed out that differences of sampling intensity and sampling methods in the two time periods may cause biased estimates. For instance, some species may not be surveyed during the investigation from 1997 to 2000 due to their extreme low population size, thus, the percent of species lost might be overestimated. Therefore, it is necessary to validate the conclusions of the study by using more data in the future. Fortunately, the State Administration of Forestry of China has completed the second nation-wide survey of these key protected vertebrates, which would provide opportunity to correct some biased estimations of this study. Besides, Infrared camera techniques are being widely used for wildlife monitoring and survey in China[57], which would provide more reliable survey data of the species occurrences in the coming years.

As discussed above, although we found the significant positive associations between species loss and increase of human population or climate change, these associations do not necessarily mean that those drivers are the direct causes of species loss or responsible for the observed patterns of species loss, instead, indirectly via increased habitat destruction or hunting by people (for instance), which need further investigation in future studies.

### Implications for biodiversity conservation

Our results provide new insights into the management and conservation of key terrestrial vertebrates in China as well other countries facing similar pressure of population increase and climate change. Based on our estimated proportion of species lost, at the county level, China has lost more than a quarter of its key terrestrial vertebrate species and almost half of its mammal species within about 5 decades. The degree of species loss is much higher than the background rate of pre-human history, and beyond this study it indicates a global problem. Thus, urgent action is needed to protect biodiversity. Because the temperature increase speed and the impacts of habitat destruction and fragmentation have accelerated in industrial times, to reduce their negative effects, it is urgent to expand size of our current protected areas. Protected areas need to either be large or to have connecting corridors between nature reserves for animals or plants to move among isolated protected areas, or to new habitats towards the poles or top of mountains for species driven by climate warming.

## Supporting information

S1 Fig **Human population density change index (A), temperature change index (B), and precipitation change index (C) of China.** The color range indicates the value of change.
(DOCX)

S2 Fig **Species richness (number of species) in 2356 counties in China for all species (A), mammals (B), birds (C) and amphibians and reptiles (D).** The color range indicates the number of species; empty area indicates missing values.
(DOCX)

S3 Fig **The diagnostics of spatial autocorrelation of residuals from the GAM models for all species (A), mammals (B), birds (C) and amphibians and reptiles (D).** The significance of the autocorrelation was assessed by 1000 permutations. The analyses were carried out in R (version 3.2.1) using the ncf package. The filled dot indicates the spatial autocorrelation is significant while the hollow dot indicates non-significant. The plots showed that there was no or very little spatial autocorrelation in the residuals from all the models.
(DOCX)

S1 Dataset The percent of species lost and species richness for all species (PSL and B), mammals (PSLm and Bm), birds (PSLb and Bb), and amphibians and reptiles (PSLa and Ba), human population density change (H), temperature change (T), precipitation change (P) and centroid longitude and latitude of 2356 counties in China. (XLSX)
