# Supplementary material for: Quantifying the effects of climate and anthropogenic change on regional species loss in China
Source: PLoS One. 2018 Jul 25;13(7):e0199735. doi: 10.1371/journal.pone.0199735 (PMC6059391; doi:10.1371/journal.pone.0199735)
Supplement: S1 Fig — Human population density change index (A), temperature change index (B), and precipitation change index (C) of China. The color range indicates the value of change. (DOCX) [file pone.0199735.s001.docx]

**
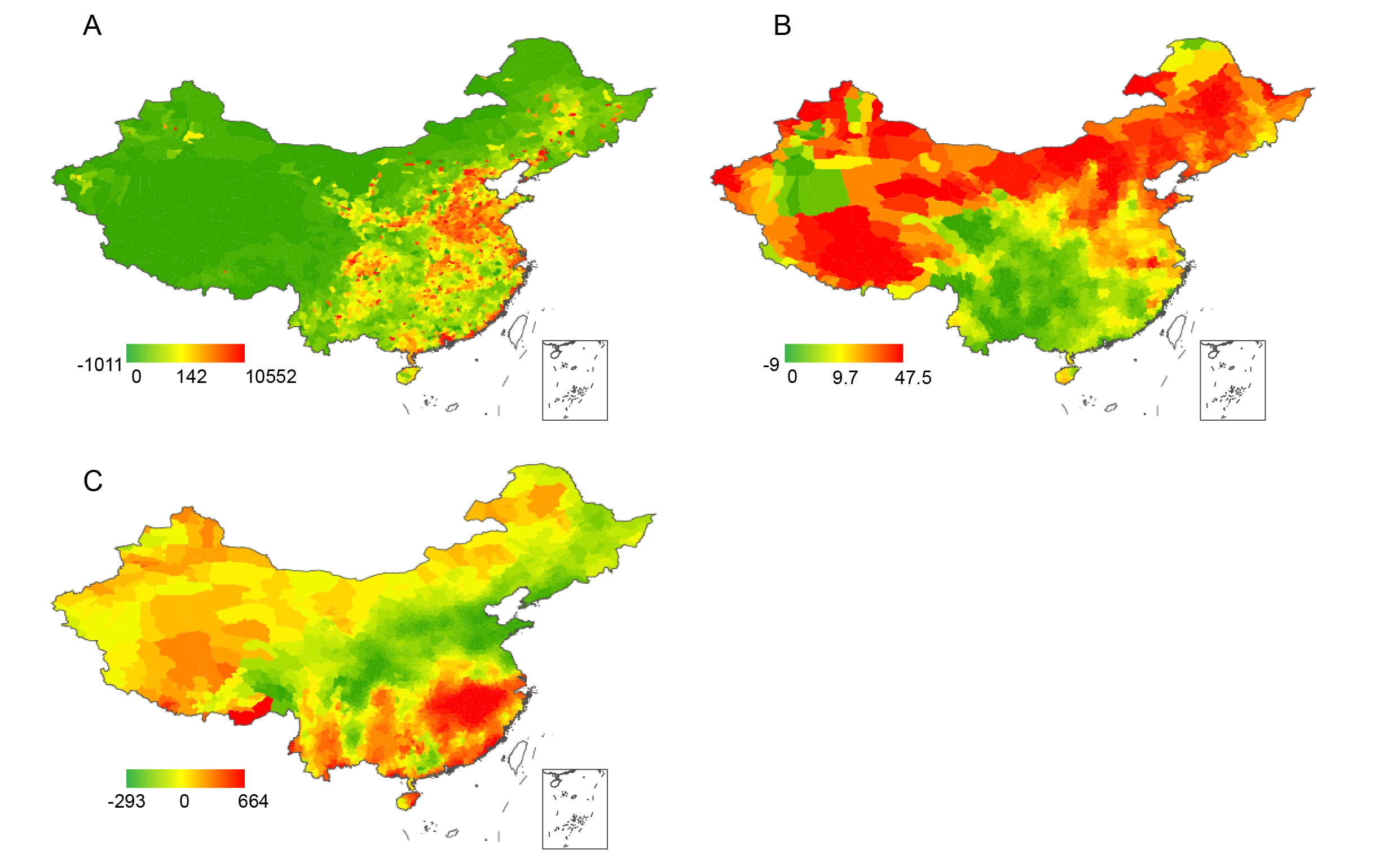
**

**S1 Fig. Human population density change index (A), temperature change index (B), and precipitation change index (C) of China.** The color range indicates the value of change.
