# Supplementary material for: Quantifying the effects of climate and anthropogenic change on regional species loss in China
Source: PLoS One. 2018 Jul 25;13(7):e0199735. doi: 10.1371/journal.pone.0199735 (PMC6059391; doi:10.1371/journal.pone.0199735)
Supplement: S2 Fig — Species richness (number of species) in 2356 counties in China for all species (A), mammals (B), birds (C) and amphibians and reptiles (D). The color range indicates the number of species; empty area indicates missing values. (DOCX) [file pone.0199735.s002.docx]

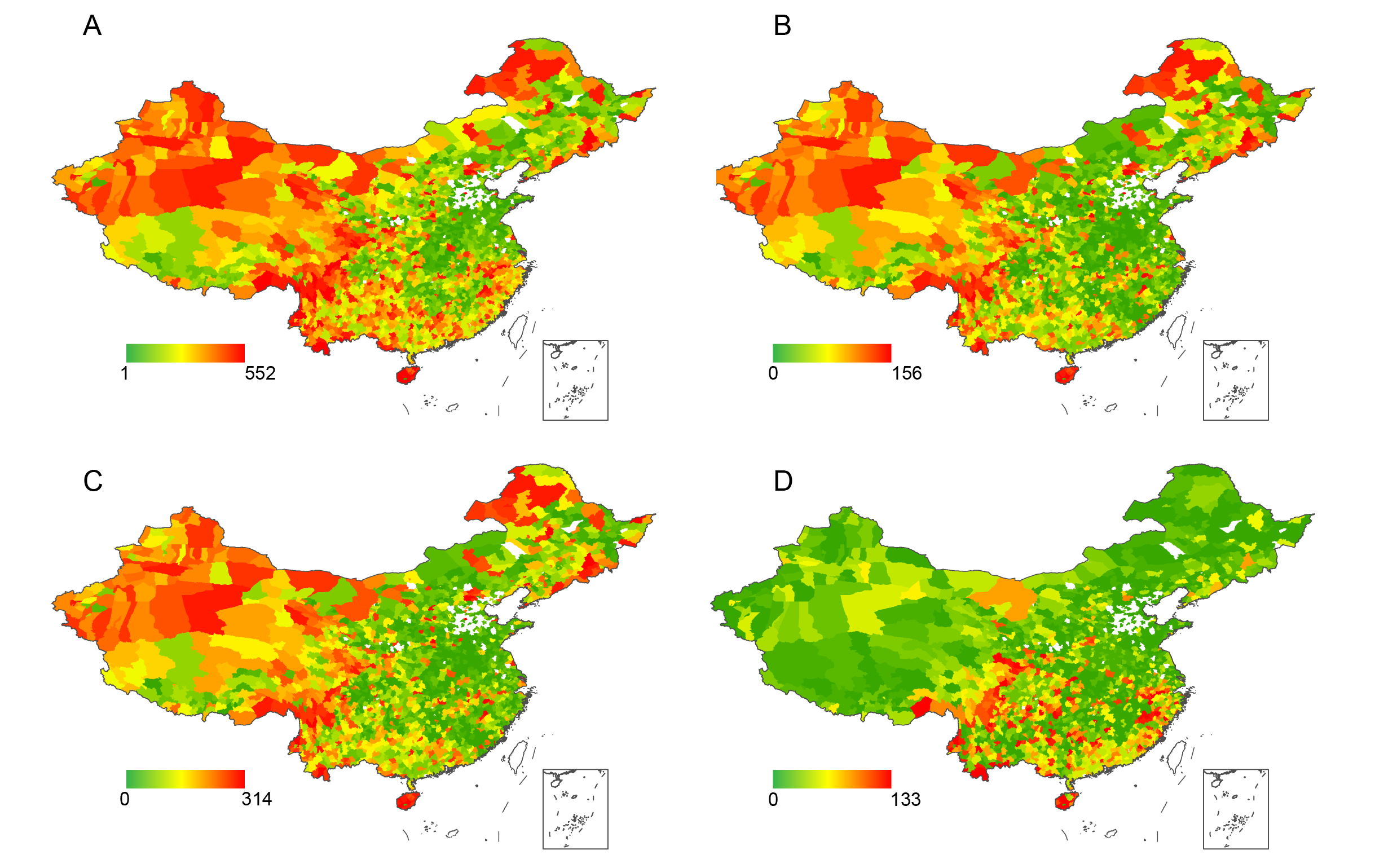


**S2 Fig. Species richness (number of species) in 2365 counties in China for all species (A), mammals (B), birds (C) and amphibians and reptiles (D).** The color range indicates the number of species; empty area indicates missing values.
