# Supplementary material for: Quantifying the effects of climate and anthropogenic change on regional species loss in China
Source: PLoS One. 2018 Jul 25;13(7):e0199735. doi: 10.1371/journal.pone.0199735 (PMC6059391; doi:10.1371/journal.pone.0199735)
Supplement: S3 Fig — The diagnostics of spatial autocorrelation of residuals from the GAM models for all species (A), mammals (B), birds (C) and amphibians and reptiles (D). The significance of the autocorrelation was assessed by 1000 permutations. The analyses were carried out in R (version 3.2.1) using the ncf package. The filled dot indicates the spatial autocorrelation is significant while the hollow dot indicates non-significant. The plots showed that there was no or very little spatial autocorrelation in the residuals from all the models. (DOCX) [file pone.0199735.s003.docx]

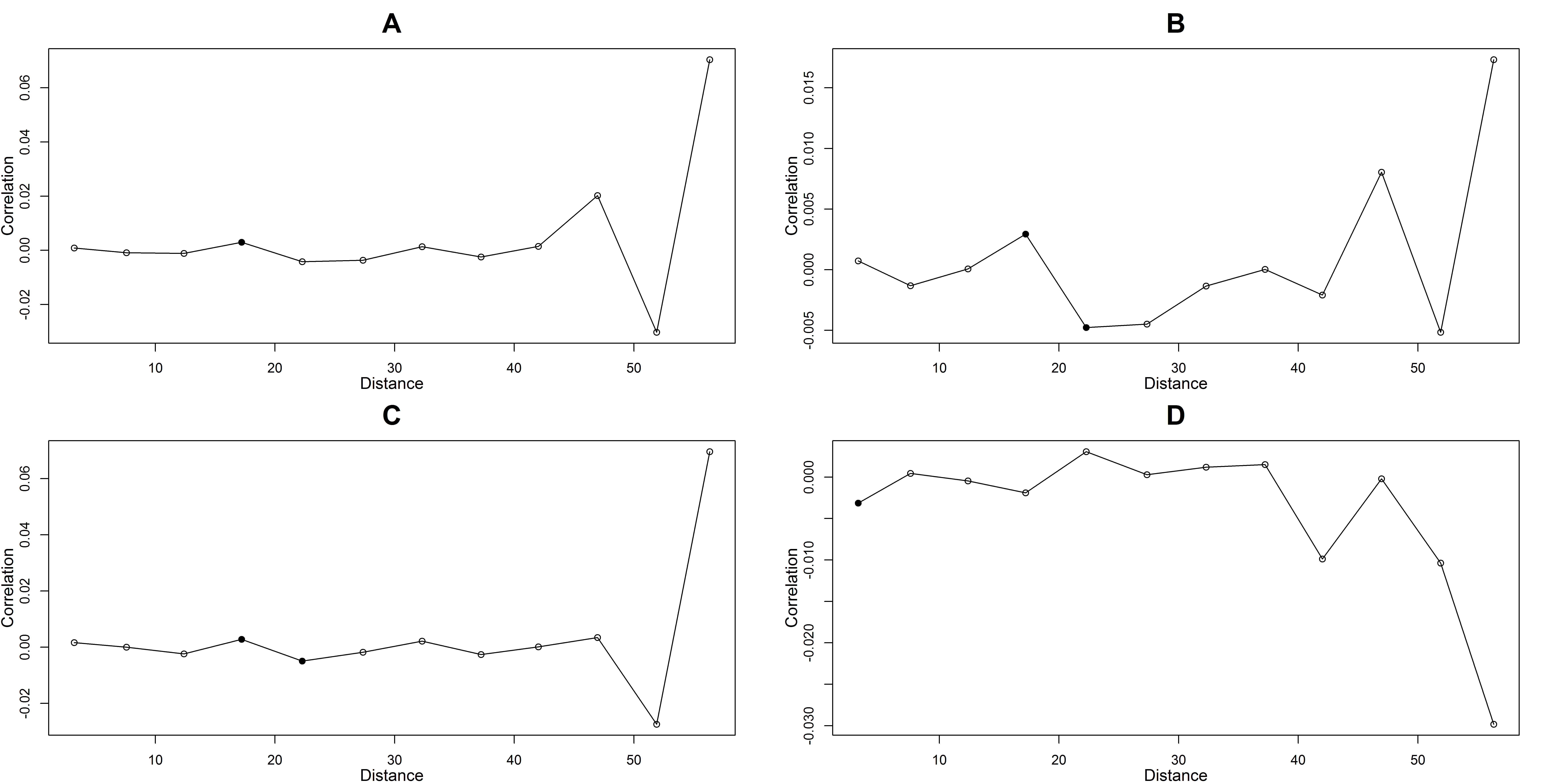


**S3 Fig. The diagnostics of spatial autocorrelation of residuals from the GAM models for all species (A), mammals (B), birds (C) and amphibians and reptiles (D).** The significance of the autocorrelation was assessed by 1000 permutations. The analyses were carried out in R (version 3.2.1) using the ncf package. The filled dot indicates the spatial autocorrelation is significant while the hollow dot indicates non-significant. The plots showed that there was no or very little spatial autocorrelation in the residuals from all the models.
